# Deletion of *IFT80* Impairs Epiphyseal and Articular Cartilage Formation Due to Disruption of Chondrocyte Differentiation

**DOI:** 10.1371/journal.pone.0130618

**Published:** 2015-06-22

**Authors:** Xue Yuan, Shuying Yang

**Affiliations:** 1 Department of Oral Biology, School of Dental Medicine, University of Buffalo, State University of New York, Buffalo, NY, United States of America; 2 Developmental Genomics Group, New York State Center of Excellence in Bioinformatics and Life Sciences, University of Buffalo, State University of New York, Buffalo, NY, United States of America; University of Alabama at Birmingham, UNITED STATES

## Abstract

Intraflagellar transport proteins (IFT) play important roles in cilia formation and organ development. Partial loss of IFT80 function leads Jeune asphyxiating thoracic dystrophy (JATD) or short-rib polydactyly (SRP) syndrome type III, displaying narrow thoracic cavity and multiple cartilage anomalies. However, it is unknown how IFT80 regulates cartilage formation. To define the role and mechanism of IFT80 in chondrocyte function and cartilage formation, we generated a *Col2α1; IFT80^f/f^* mouse model by crossing *IFT80^f/f^* mice with inducible *Col2α1-CreER* mice, and deleted *IFT80* in chondrocyte lineage by injection of tamoxifen into the mice in embryonic or postnatal stage. Loss of *IFT80* in the embryonic stage resulted in short limbs at birth. Histological studies showed that *IFT80-*deficient mice have shortened cartilage with marked changes in cellular morphology and organization in the resting, proliferative, pre-hypertrophic, and hypertrophic zones. Moreover, deletion of *IFT80* in the postnatal stage led to mouse stunted growth with shortened growth plate but thickened articular cartilage. Defects of ciliogenesis were found in the cartilage of *IFT80*-deficient mice and primary *IFT80*-deficient chondrocytes. Further study showed that chondrogenic differentiation was significantly inhibited in *IFT80*-deficient mice due to reduced hedgehog (Hh) signaling and increased Wnt signaling activities. These findings demonstrate that loss of *IFT80* blocks chondrocyte differentiation by disruption of ciliogenesis and alteration of Hh and Wnt signaling transduction, which in turn alters epiphyseal and articular cartilage formation.

## Introduction

Primary cilium, first described decades ago, is now considered to be a critical organelle in the regulation of organ development and function [1, 2]. Almost all vertebrate cells have primary cilia [1, 3]. Those microtubule-based structures protrude from the cell surface, sense environment changes and transduce intercellular signaling [2, 4]. In humans, mutations with cilia structural loss or functional defects lead to serious diseases with severe skeletal abnormalities [5, 6]. The first evidence showing the presence of primary cilia in the skeleton was found about 40 years ago with the discovery of cilia on chondrocytes [7, 8]. Later studies showed that cilia participate in almost every aspect of chondrocyte biology, including differentiation, biomechanical signal transduction, endocytosis, osmotic response, and apoptosis [9].

Since primary cilia are important in development, extensive studies have been done recently to uncover their structure and associated proteins [10, 11]. It is clear now that construction and function of cilia requires effective intraflagellar transport (IFT), which is a bidirectional transport system operated by IFT protein complexes and IFT motors [4]. IFT protein complexes, divided into complex A and complex B, contain 20 IFT proteins. IFT complex A is in charge of retrograde transport (from cilia tip to cytosol), while IFT complex B is involved in anterograde transport (from cytosol to cilia tip). Mutations of some IFT proteins, such as *IFT88* [12, 13], *IFT172* [14], and *IFT122* [15], cause cilia loss.

IFT80 is a core protein in IFT complex B. Loss of *IFT80* reduces cilia number in zebrafish, or results in shortened cilia or cilia loss in Tetrahymena thermophila [16, 17]. Our previous studies showed silence of *IFT80* caused shortened cilia or cilia loss in mesenchymal progenitor cell line C3H10T1/2 and bone marrow derived stromal cells (BMSCs) [18, 19]. Mutations of *IFT80* in human have been identified in Jeune asphyxiating thoracic dystrophy (JATD) [16] and short-rib polydactyly (SRP) syndrome type III [20]. Patients suffering from these diseases display narrow thoracic cavity and multiple cartilage anomalies, suggesting that IFT80 is involved in chondrocyte differentiation and function. However, the role of IFT80 in chondrocyte development and cartilage formation *in vivo* remains undefined. Recently, Rix et al., generated a hypomorphic IFT80 knockout mouse model with low-level wild type *IFT80* transcript production and found this partial deletion of *IFT80* caused 98% embryonic lethal [21]. About 2% homozygotes could survive to postnatal stage. Those mice displayed growth retardation and constriction of the rib cage similar to the phenotype of JATD and SRP type III, suggesting IFT80 plays a role in chondrocyte development and function. However, this IFT80 trap-line is hypomorphic rather than a true null, due to low-level wild type *IFT80* transcript production. Moreover, only about 2% mutant mice could survive, which makes it difficult to study the exact role of IFT80 in chondrocyte lineage.

To address this issue, we used *Col2α1-CreER* mice to delete *IFT80* in the chondrocyte lineage in this study [22]. Cre activity in chondrocyte lineage is induced by administration of tamoxifen in this *Col2α1-CreER* line, allowing us to study the role of IFT80 in cartilage development in both embryonic and postnatal stages. We found that embryonic deletion of *IFT80* in chondrocytes resulted in cilia loss and chondrodysplasia, and postnatal deletion of *IFT80* reduced the growth plate length but thickened articular cartilage in *Col2α1; IFT80* ^*f/f*^ mice. We further found that deletion of *IFT80* in primary chondrocytes caused cilia loss, accompanying with significantly inhibition of chondrogenic differentiation, disruption of Hh signaling, and up-regulation of Wnt signaling. Our findings suggest that altered Hh and Wnt signaling contribute to the cartilage defects and the dwarfish phenotype in the *Col2α1; IFT80* ^*f/f*^ mice.

## Methods

### Mice and tamoxifen injection

*IFT80* ^*f/f*^ mouse model, which has two LoxP sites flanking exon 6 of *IFT80*, has been generated as descripted in S1 Fig *Col2α1-CreER* mouse was purchased from the Jackson Laboratory (Bar Habor, ME, USA), which has strong tamoxifen-inducible *Cre* expression primarily in chondrogenic lineage cells (cartilage) [22]. *IFT80* ^*f/f*^ mice were bred with *Col2α1-CreER* mice to produce *Col2α1; IFT80* ^*f/+*^ mice. *Col2α1; IFT80* ^*f/+*^ mice were mated with each other to generate *Col2α1; IFT80* ^*f/f*^ mice. Without tamoxifen injection, *Col2α1; IFT80* ^*f/f*^ mice were indistinguishable from wild type and *IFT80* ^*f/f*^ mice. *IFT80* ^*f/f*^ mice were then crossed with *Col2α1; IFT80* ^*f/f*^ mice to produce *IFT80* ^*f/f*^ (used as control) and *Col2α1; IFT80* ^*f/f*^ mice. The pregnant females and neonatal mice were injected with tamoxifen to delete *IFT80* in the chondrogenic lineage at different stages.

Tamoxifen (Sigma, T5648) solution preparation and administration were performed as previously described with slight modification [22, 23]. Tamoxifen was first dissolved in 100% ethanol at a concentration of 100 mg/mL and then diluted with sterile corn oil to a final concentration of 20 mg/mL. Pregnant female *Col2α1; IFT80* ^*f/f*^ mice breeding with *IFT80* ^*f/f*^ were injected with 6 mg tamoxifen at 14.5, 16.5, and 18.5 days postcoitus and the newborn pups were harvested at birth. For neonatal injections, both *Col2α1; IFT80* ^*f/f*^ and *IFT80* ^*f/f*^ mice were given same dose of tamoxifen to avoid the possible side effects of tamoxifen. Four consecutive intraperitoneal injections of 1 mg tamoxifen were performed at postnatal day 4–7, and another four consecutive intraperitoneal injections of 2 mg tamoxifen were administered at postnatal day 14–17. The mice were harvested at postnatal day 30. Four to six independent litters were analyzed. And the *Col2α1; IFT80* ^*f/f*^ mice were compared to *IFT80* ^*f/f*^ mice in the same litter.

The efficiency of *IFT80* deletion was confirmed by Western blot. Cartilages from *IFT80* ^*f/f*^ and *Col2α1; IFT80* ^*f/f*^ mice were harvested and homogenized with RIPA buffer (50 mM Tris, 150 mM NaCl, 1% Triton X-100, 0.1% SDS, and 1% sodium deoxycholate) containing protease inhibitor cocktail (Sigma, St. Louis, MO). Rabbit anti-IFT80 antibody (1:400, Sigma, SAB2107274) was used to detect IFT80 expression. Goat anti-GAPDH (1:4000, Genscript) was used as internal control. This experiment was run in triplicate.

All mouse studies and procedures were conducted with approval by Institutional Animal Care and Use Committee (IACUC) of University at Buffalo.

### Histology, skeleton staining, Alizarin red staining, Von Kossa staining and Safranin O staining

Mice tibias (n = 3) were excised, fixed with 10% natural buffered formalin, and decalcified in 10% ethylenediaminetetraacetic acid (EDTA) for one to two weeks at 4°C. The samples were embedded in paraffin, sectioned at 5 μm, and stained with H&E. Quantification of cartilage length was done with Image J (NIH, Bethesda, MD, USA).

Alizarin Red/Alcian Blue staining was used to stain the whole skeleton as reported before [24–26]. Briefly, skeleton of newborn mice (n = 3) were fixed with 90% ethanol, and then stained with 0.01% Alcian Blue solution and 1% Alizarin Red S solution, respectively. Stained skeletons were stored in glycerol.

Deparaffinized slides (n = 3) were stained with 2% Alizarin red (pH = 4.2) for 2 min and then dehydrated with acetone, acetone-xylene (1:1), and xylene. Von Kossa staining was performed with 1% silver nitrate solution in glass coplin jar placed under ultraviolet light for 20–30 minutes [27]. Un-reacted silver was washed with 5% sodium thiosulfate. Fast green was used as counter stain. The slides were dehydrated with graded alcohols and mounted with permanent mounting medium. Bone volume (BV) and tissue volume (TV) were measured using Image J software with Bone J plugin [28].

Safranin O staining was used to visualize cartilage and access the content of proteoglycan. Deparaffinized slides were stained with Weigert’s iron hematoxylin and fast green, and then stained with 0.1% safranin O solution.

### Culture and differentiation of primary chondrocytes

Procedures of primary chondrocytes isolation were modified from previously published methods [29, 30]. Littermate *IFT80* ^*f/f*^ and *Col2α1; IFT80* ^*f/f*^ mice were injected with tamoxifen (1 mg/day) at postnatal day 4–7. Then, at postnatal day 10, the mice were harvested and femoral heads, femoral condyles, and tibia plateaus from these mice were isolated (soft tissue and bones were excluded). The articular cartilages were cut into pieces and then incubated with collagenase type 4 (Worthington, Lakewood, NJ) solution (3 mg/mL) for 45 min at 37°C. The cartilage pieces were washed and incubated in 0.5 mg/mL collagenase type 4 solution (diluted with DMEM+10% FBS) overnight at 37°C. Cells were collected, washed with PBS and seeded at a density of 8 × 10^3^ cells per cm^2^ in a new petri dish. Cells from at least three mice per genotype were pooled each time.

To induce differentiation, chondrocytes were cultured in chondrogenic media (DMEM+10% FBS) with the presence of ITS supplement (insulin-10 μg/mL, transferrin-5.5 μg/mL, and sodium selenite-5 ng/mL, Sigma-13146) for 3 weeks [31, 32]. The medium was changed every three days. Alcian blue staining was performed at the end of differentiation. Briefly, cells were fixed with 4% glutaraldehyde for 15 min, washed, and stained with 1% Alcian blue in 3% acetic acid (pH = 2.5) for 2 hours. Then, the cells were washed with 3% acetic acid three times before taking images. For quantitative analysis, dye from each well was extracted with 150 μL of 6M guanidine-HCl for 2h. The extracted dyes were transferred to 96-well plates and measured at 620 nm [19]. This experiment was run in triplicate and repeated three times with different cells.

### Reporter assay

To explore potential abnormalities of Hh signaling in *IFT80*-deficiency chondrocytes, primary chondrocytes derived from postnatal day 10 *IFT80* ^*f/f*^ and *Col2α1; IFT80* ^*f/f*^ mice (received tamoxifen from E14.5, E16.5 and E18.5) were subjected to reporter assay as we described previously [19]. Briefly, 1×10^6^ cells were co-transfected with the constructs of 3 μg 8 × Gli-Luc (gift from Dr. Fernandez- Zapico [33]), and 0.6 μg pRL-TK Renilla (Promega, Madison, WI) with Fugene HD (Promega). Renilla luciferase was used as internal control. Cells were induced with chondrogenic media for 3 days, and then treated with 1 μg/mL recombinant mouse Shh N-terminus (R&D systems, Minneapolis, MN) for 8 h [34].

To test Wnt signaling pathway activity, 1×10^6^ chondrocytes were transiently co-transfected with internal control pRL-TK Renilla (Promega) and either 3 μg M50 Super 8×TOPFlash vector (Addgene, Cambridge, MA), or 3 μg M51 Super 8×FOPFlash (TOPFlash mutant, used as control). Cells were then induced with chondrogenic media for 3 days, and stimulated with 100 ng/mL recombinant Wnt3a (Applied stem cell, Menlo Park, CA) for 8 h [19].

The luciferase activities in cell lysates were measured by Veritas Microplate Luminometer (Turner Biosystem, Sunnyvale, CA) with the Dual-luciferase reporter assay kit (Promega) following the manufacturer’s instructions.

The experiment was run in triplicate and repeated three times. The data are means from one representative experiment.

### qPCR

Total RNA was extracted from cultured primary chondrocytes with Trizol (Invitrogen, Carlsbad, CA) following the manufacturer’s instructions. cDNA was synthesized from 2 μg total RNA with RNA to cDNA EcoDry Premix kit (Clontech, Palo Alto, CA). qPCR was performed with SYBR Green PCR master Mix (Invitrogen) on the ABI PRISM 7500 real time PCR machine (Invitrogen). All qPCR were run in triplicate and normalized to the expression of GAPDH. The calculation of relative expression was performed according to the 2^−ddCT^ method [35]. Each reaction was run in triplicate and independently repeated three times.

Sequences and product lengths for each primer pair were as follow: IFT80 (Forward: 5’-AAGGAACCAAAGCATCAAGAATTAG-3’; Reverse: 5’-AGATGTCATCAGGCAGCTTGAC-3’; 148 bp); Sox9 (Forward: 5’-TCCCCGCAACAGATCTCCTA -3’; Reverse 5’- AGGTGGAGTAGAGCCCTGAG -3’; 157 bp); Aggrecan (Forward: 5’- CGTTGCAGACCAGGAGCAAT -3’; Reverse 5’- AGGAGTGACAATGCTGCTCA -3’; 145 bp); Type X collagen (Forward: 5’-CGGTACCAAACGCCCACAGGC -3’; Reverse 5’-GCCTGGCTTCCCCGTGGCTGATAT-3’; 258 bp); and GAPDH (Forward: 5’- CACATTGGGGGTAGGAACAC -3’; Reverse 5’- AACTTTGGCATTGTGGAAGG -3’; 222 bp).

### Cilia staining

Immunofluorescence staining was performed with anti-acetylated α-tubulin (Sigma) to visualize cilia structure [19]. Fixed chondrocytes or deparaffinized tibia sections were incubated with primary acetylated α-tubulin antibody (1:500, sigma) overnight at 4°C and then stained with Alexa Fluor 647 conjugated anti-mouse IgG (Invitrogen) antibody. DAPI (6-diamidino-2-phenylindole, Sigma) staining was used as a counterstain of nuclei. The images were taken with an Zeiss Axio imager microscope (Carl Zeiss, Germany). Each experiment was independently repeated at least three times.

### Statistical analysis

Statistical analysis was performed using Student's t-test for comparison between two groups or two-way ANOVA followed by Bonferroni’s post hoc tests for grouped samples. A p-value of <0.05 was considered to be significant. The results were expressed as the mean ± SEM.

## Results

### Embryonic deletion of *IFT80* in chondrocytes resulted in short limbs

**Fig 1:**
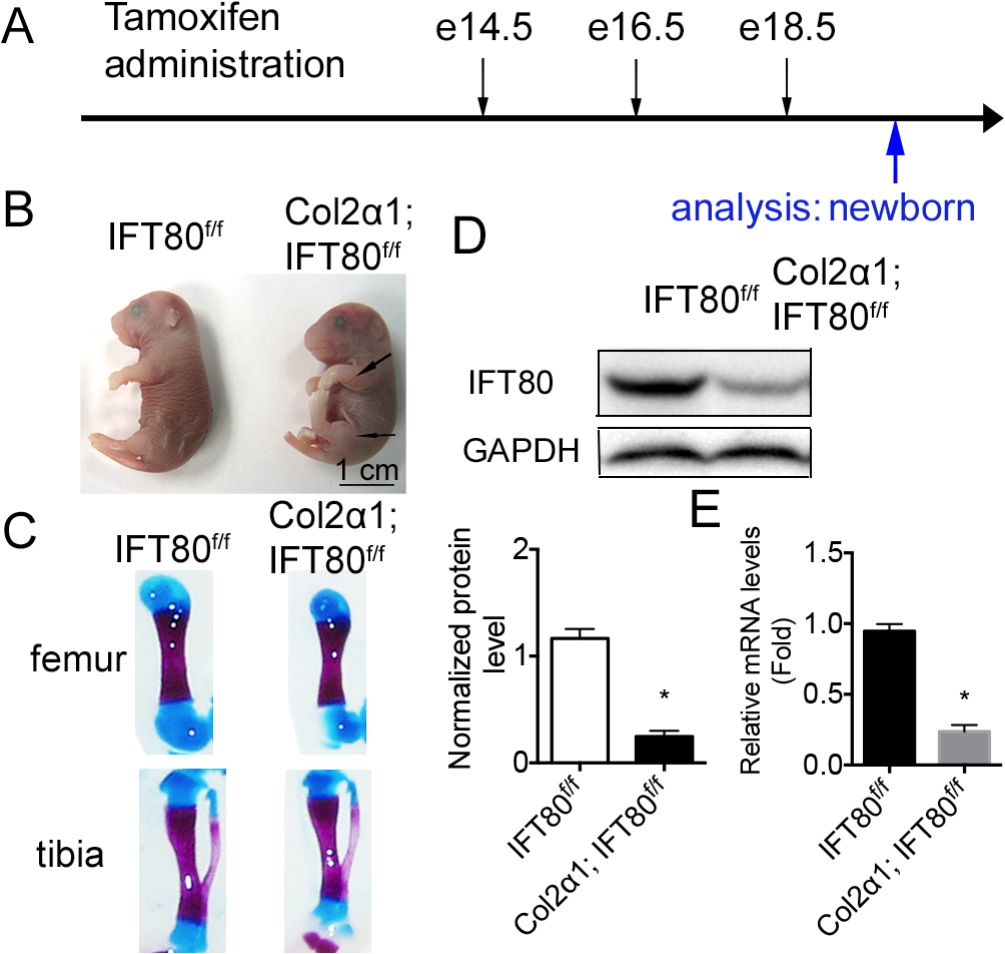
Deletion of *IFT80* in the embryonic stage. (A) Line drawing showing the timing of tamoxifen administrations for *IFT80* deletion in embryonic stage. Arrows above the line indicate the time of tamoxifen administration to the pregnant females at 14.5, 16.5, and 18.5 days postcoitus. The blue arrow below the line indicates the harvest time. (B) Image of *IFT80* ^*f/f*^ and *Col2α1; IFT80* ^*f/f*^ newborn mice exposed to tamoxifen at E14.5, E16.5, and E18.5. Arrows indicate the shortened limbs. (C) Alizarin red and Alcian blue staining of hindlimbs of *IFT80* ^*f/f*^ and *Col2α1; IFT80* ^*f/f*^ newborn mice. (D) Western blot analysis of IFT80 expression in the cartilage of *IFT80* ^*f/f*^ and *Col2α1; IFT80* ^*f/f*^ mice. IFT80 protein level was normalized to GAPDH (n = 3, *P<0.001, significantly different from the *IFT80* ^*f/f*^ group). (E) qPCR of *IFT80* expression. *IFT80* expression level was normalized to GAPDH (n = 3, *P<0.001, significantly different from the *IFT80* ^*f/f*^ group).

To study the role of IFT80 in cartilage development in the embryonic stage, *IFT80* ^*f/f*^ mice were mated with *Col2α1; IFT80* ^*f/f*^ mice. Pregnant mothers were injected with tamoxifen at the indicated times shown in Fig 1A. Newborn mice were harvested for the phenotype analysis. *Col2α1; IFT80* ^*f/f*^ mice displayed shorted limbs (Fig 1B). Alizarin Red and Alcian blue staining of hindlimbs confirmed both tibias and femurs from *Col2α1; IFT80* ^*f/f*^ mice were shorter than those from *IFT80* ^*f/f*^ mice (Fig 1C). Western blot and qPCR confirmed that IFT80 expression was deleted in the cartilage of *Col2α1; IFT80* ^*f/f*^ newborn mice (Fig 1D and 1E).

### Embryonic deletion of *IFT80* in chondrocytes resulted in chondrodysplasia

**Fig 2:**
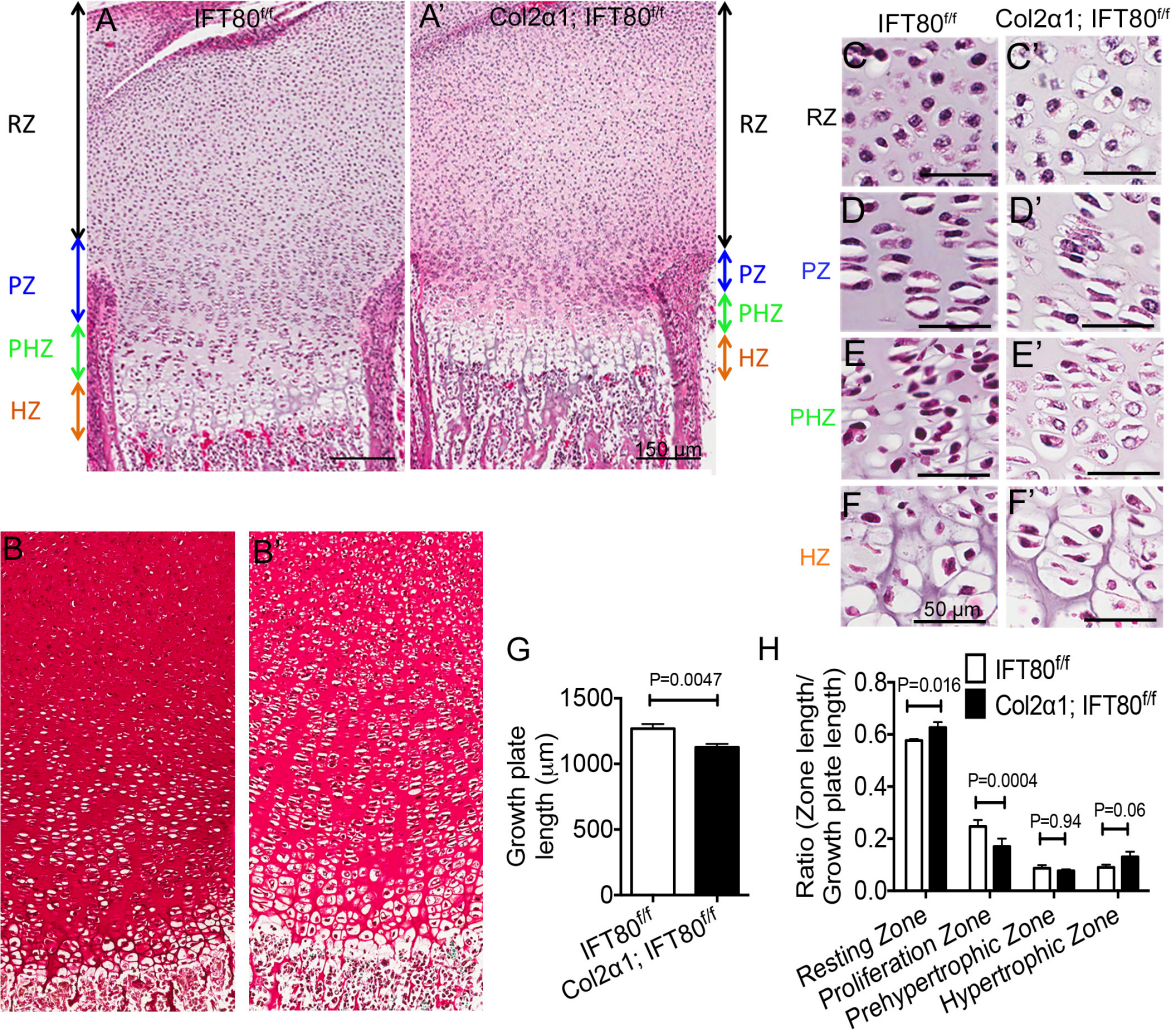
Histological examination of the tibial growth plates of newborn *IFT80* ^*f/f*^ mice and *Col2α1; IFT80* ^*f/f*^ mice. (A and A’) Histological examination of tibial growth plates of newborn *IFT80* ^*f/f*^ mice (A) and *Col2α1; IFT80* ^*f/f*^ mice (A’) by H&E staining. Mice were exposed to tamoxifen at E14.5, E16.5, and E18.5. The growth plate can be divided transversely into resting zone (RZ), proliferation zone (PZ), prehypertrophic zone, (PHZ) and hypertrophic zone (HZ). (B and B’) Safranin O staining of tibial growth plates of newborn *IFT80* ^*f/f*^ mice (B) and *Col2α1; IFT80* ^*f/f*^ mice (B’). (C-F and C’-F’) Histological examination of chondrocytes morphology in higher magnification view. Chondrocytes in the resting zone of *Col2α1; IFT80* ^*f/f*^ mice (C’) displayed hypercellularity compared to those in *IFT80* ^*f/f*^ mice (C). Cells were less organized in the proliferation zone of *Col2α1; IFT80* ^*f/f*^ mice (D’) compared to those in *IFT80* ^*f/f*^ mice (D). In the prehypertrophic and hypertrophic zones, chondrocytes were larger, with less cell density, in *Col2α1; IFT80* ^*f/f*^ mice (E’ and F’) than those in *IFT80* ^*f/f*^ mice (E and F). (G) Quantitative analysis of cartilage length (n = 3). (H) Quantitative analysis of each zone’s lengths. Data was reported as a ratio of the zone length to the total growth plate length (n = 3).

To further investigate the structure of the cartilage, histological examination was performed on the tibias from *IFT80* ^*f/f*^ mice and *Col2α1; IFT80* ^*f/f*^ mice. Chondrocytes displayed different morphology within different zones, which including resting, proliferation, prehypertrophic, and hypertrophic zones in epiphyseal plate (Fig 2A). *Col2α1; IFT80* ^*f/f*^ mice showed disorganized cartilage (Fig 2A’). Safranin O staining further confirmed the abnormal cartilage structure in *Col2α1; IFT80* ^*f/f*^ mice and showed the reduced proteoglycans production in *Col2α1; IFT80* ^*f/f*^ mice (Fig 2B and 2B’). Cartilages from *IFT80* ^*f/f*^ group displayed the typical and clear demarcation of layers with distinct cellular morphology (Fig 2C–2F). The chondrocytes were small and round- or oval-shaped in the resting zone (Fig 2C). However, the resting chondrocytes in *Col2α1; IFT80* ^*f/f*^ displayed hypercellularity compared to those in *IFT80* ^*f/f*^ mice (Fig 2C’). In the proliferation zone of *IFT80* ^*f/f*^ mice, the chondrocytes were flattened and organized into linear clusters parallel to the long bone, representing cells that have recently divided (Fig 2D). However, the proliferation zone was less organized and some of the cells were distributed singularly in *Col2α1; IFT80* ^*f/f*^ mice (Fig 2D’). Additionally, in *IFT80* ^*f/f*^ mice, the cells in the prehypertrophic and hypertrophic zones were large and vacuolated (Fig 2E and 2F), and the clear areas of cytoplasm indicate the lipid droplets and glycogen stores. But in the prehypertrophic zone and hypertrophic zones of *Col2α1; IFT80* ^*f/f*^ group, chondrocytes were larger but had smaller nuclei; moreover, the cell density was also reduced (Fig 2E’ and 2F’) compared to that in *IFT80* ^*f/f*^ mice (Fig 2E and 2F).

Deletion of *IFT80* in chondrocytes during the embryonic stage significantly shortened the cartilage length (Fig 2A, 2A’, 2B, 2B’, 2G and 2H). The length of the growth plate in *IFT80* ^*f/f*^ mice was 1269 ± 19.9 μm, whereas the length in *Col2α1; IFT80* ^*f/f*^ mice was 1127 ± 15.1 μm (Fig 2G). The most significant shortened zone was proliferation zone (Fig 2H), which decreased from 313 ± 22.6 μm (*IFT80* ^*f/f*^ mice) to 190 ± 19.8 μm in *Col2α1; IFT80* ^*f/f*^ mice (p<0.0001).

### Embryonic deletion of *IFT80* in chondrocytes inhibited endochondral bone ossification

**Fig 3:**
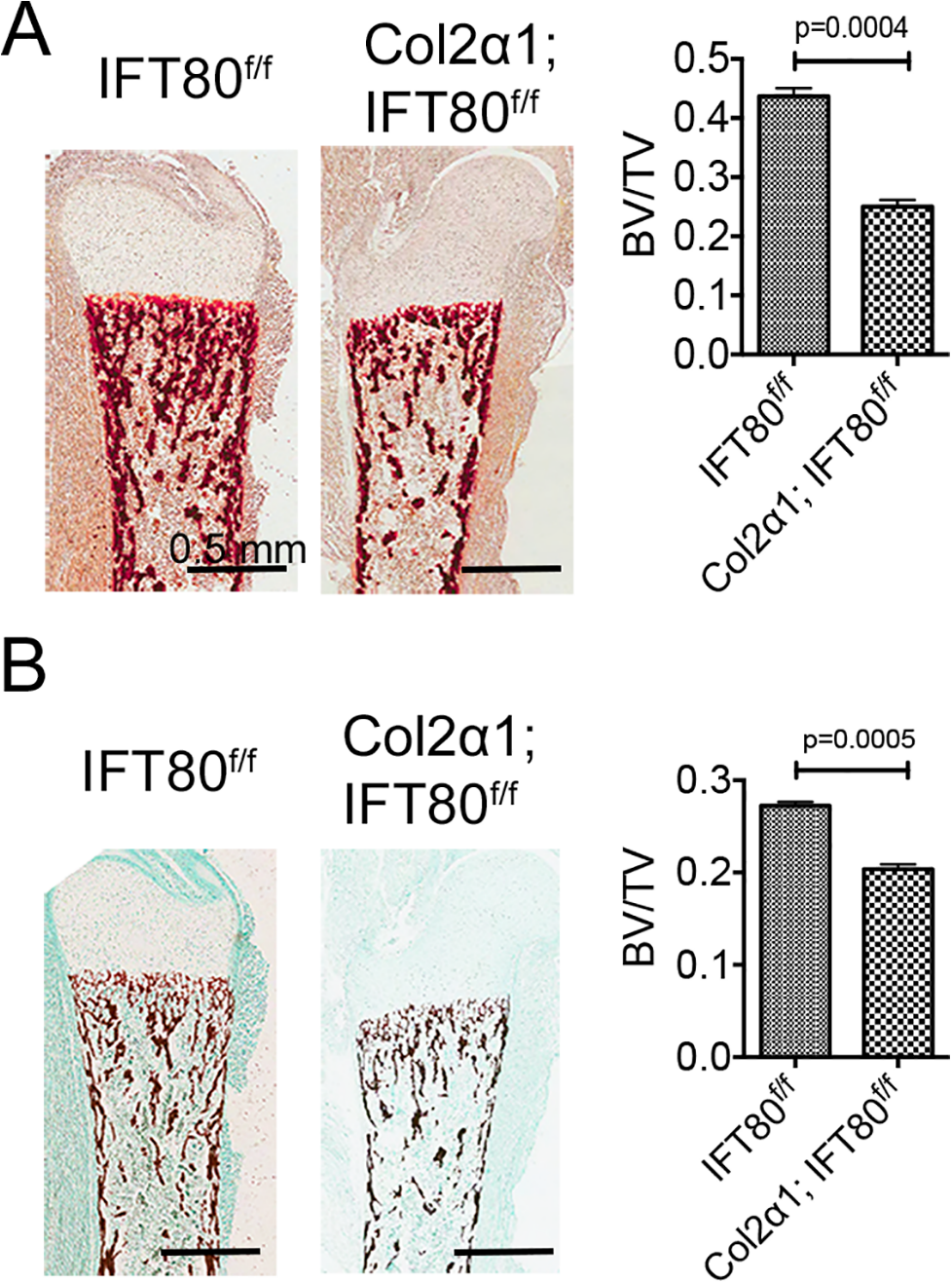
Endochondral bone ossification was examined of the tibial growth plates of newborn *IFT80* ^*f/f*^ mice and *Col2α1; IFT80* ^*f/f*^ mice using Alizarin Red/Von Kossa staining. Mice exposed to tamoxifen at E14.5, E16.5, and E18.5. (A) Alizarin Red staining of the tibial section. Bone volume (BV) and tissue volume (TV) were quantified using Image J (n = 3). (B) Von Kossa staining of the tibial section. Fast green was used as a counter stain. BV/TV were quantified using Image J (n = 3).

To further study the role of IFT80 in endochondral bone formation, we performed histological analysis, Alizarin red staining and Von Kossa staining to analyze endochondral bone ossification. We found the trabecular bone volume was decreased in *Col2α1; IFT80* ^*f/f*^ mice (S2 Fig). Additionally, there was no significant difference in the mineralization capability of periochondal cells, as the bone collar formation was not affected (Fig 3A and 3B). However, the bone matrix and mineralization, as well as the trabecular bone volume, were dramatically decreased in *Col2α1; IFT80* ^*f/f*^ mice (Fig 3A and 3B). These results suggest that IFT80 is required for chondrogenesis and endochondral bone formation in the embryonic stage.

### Postnatal deletion of *IFT80* resulted in growth retardation in *Col2α1; IFT80* ^*f/f*^

**Fig 4:**
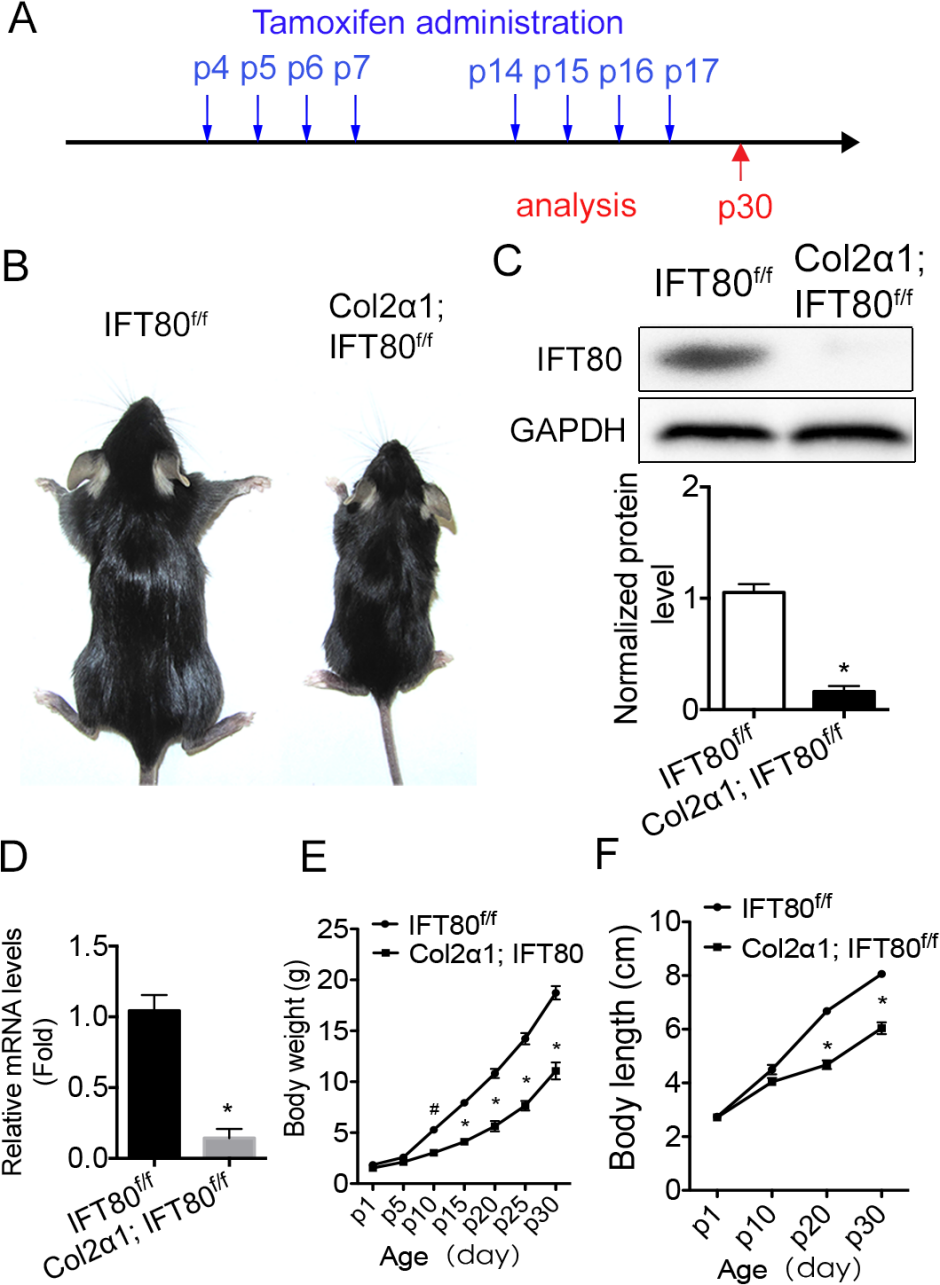
Deletion of *IFT80* in the postnatal stage. (A) Line drawing showing the timing of tamoxifen administration for *IFT80* deletion in postnatal stage. Arrows above the line indicate the time of tamoxifen injection at P4-7 and P14-17. The blue arrow below the line indicates the harvest time P30. (B) Image of *IFT80* ^*f/f*^ and *Col2α1; IFT80* ^*f/f*^ P30 mice, which were administered tamoxifen at P4-7 and P14-17. (C) Western blot analysis of IFT80 expression in the cartilage of *IFT80* ^*f/f*^ and *Col2α1; IFT80* ^*f/f*^ mice at P30. IFT80 protein level was normalized to GAPDH (n = 3, *P<0.001, significantly different from the *IFT80* ^*f/f*^ group). (D) qPCR of *IFT80* expression. IFT80 expression level was normalized to GAPDH (n = 3, *P<0.001, significantly different from the *IFT80* ^*f/f*^ group). (E) The average body weight of tamoxifen-injected *IFT80* ^*f/f*^ and *Col2α1; IFT80* ^*f/f*^ mice (n = 6, #p = 0.0022, *p<0.0001, significantly different from the *IFT80* ^*f/f*^ group). (F) The average body length of tamoxifen-injected *IFT80* ^*f/f*^ and *Col2α1; IFT80* ^*f/f*^ mice (n = 6, *p<0.0001, significantly different from *IFT80* ^*f/f*^ group).

To further study the role of IFT80 in the postnatal stage, mice were administered tamoxifen at P4-6 and P14-17 to delete *IFT80* in cartilage, and analyzed at P30 (Fig 4A). Both *Col2α1; IFT80* ^*f/f*^ and *IFT80* ^*f/f*^ mice (from same litter) were injected with same amount tamoxifen to eliminate any side effects from tamoxifen injection. Tamoxifen-injected *Col2α1; IFT80* ^*f/f*^ mice were significantly smaller than tamoxifen-injected *IFT80* ^*f/f*^ mice at P30 (Fig 4B). They showed normal activity and were able to compete for lactation in the early stage of development, and consume water and food after wean. Cartilage from P30 tamoxifen-injected *Col2α1; IFT80* ^*f/f*^ mice showed significantly decreased IFT80 expression (*P<0.0001), confirming that postnatal injection of tamoxifen successfully deleted *IFT80* in the chondrocyte lineage (Fig 4C and 4D). Further study found that *Col2α1; IFT80* ^*f/f*^ mice had significant lower body weight starting from P10 (Fig 4E) and shorter body length starting from P20 (Fig 4F).

### Postnatal deletion of *IFT80* caused shortened growth plate but thickened articular cartilage in *Col2α1; IFT80* ^*f/f*^ mice

**Fig 5:**
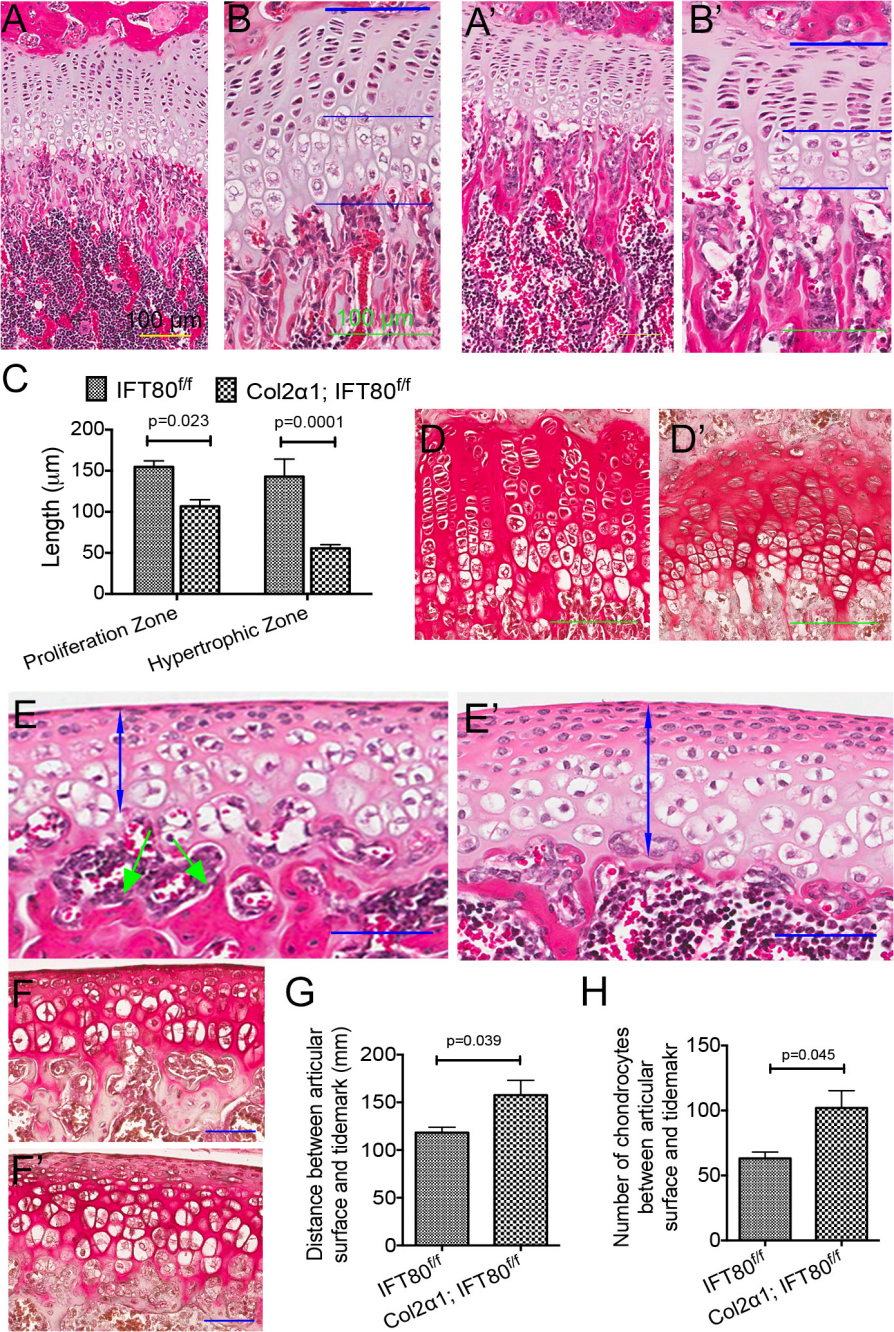
Histological examination of tibial growth plates of P30 mice. Mice were administered tamoxifen at P4-7 and P14-17. (A-B and A’-B’) H&E staining of tibial growth plates of P30 *IFT80* ^*f/f*^ mice (A—B) and *Col2α1; IFT80* ^*f/f*^ mice (A’—B’). (C) Quantitative analysis of the length of the proliferation zone and the hypertrophic zone. The length of the proliferation zone in *Col2α1; IFT80* ^*f/f*^ mice was significantly reduced compared to *IFT80* ^*f/f*^ mice (n = 3). (D and D’) Safranin O stained tibial growth plates of P30 *IFT80* ^*f/f*^ mice (D) and *Col2α1; IFT80* ^*f/f*^ mice (D’). (E and E’) H&E staining of articular cartilage of P30 *IFT80* ^*f/f*^ mice (E) and *Col2α1; IFT80* ^*f/f*^ mice (E’). The green arrows indicate the ossified bone, and the blue double-ended arrows measure the distance between the tidemark and the surface of the articular cartilage. (F and F’) Safranin O stained articular cartilage of P30 *IFT80* ^*f/f*^ mice (F) and *Col2α1; IFT80* ^*f/f*^ mice (F’). (G) Quantification of the distance between the articular surface and the tidemark in the tibias of *IFT80* ^*f/f*^ and *Col2α1; IFT80* ^*f/f*^ mice (n = 3). (H) Quantification of the number of chondrocytes in the articular cartilage between the articular surface and tidemark (40× magnification fields) (n = 3).

To further investigate how IFT80 regulates growth plate development, we examined the structure of tibia growth plates by histological analysis. Consistent with the dwarfish phenotype, *Col2α1; IFT80* ^*f/f*^ mice displayed a significantly shorter proliferation zone and a slightly shorter hypertrophic zone compared to *IFT80* ^*f/f*^ mice (Fig 5A, 5A’, 5B, 5B’ and 5C). Safranin O staining showed the disorganized growth plate structure and reduced proteoglycan production (Fig 5D and 5D’).

We also compared the articular cartilage thicknesses and found that the morphology of the articular cartilage was altered in the tibia of *Col2α1; IFT80* ^*f/f*^ mice (Fig 5E, 5E’, 5F and 5F’). The distance between the articular surface and the tidemark was also significantly increased in the tibia of *Col2α1; IFT80* ^*f/f*^ mice (Fig 5G). Furthermore, the number of chondrocytes in the articular cartilage above the tidemark was significantly greater in *Col2α1; IFT80* ^*f/f*^ mice (Fig 5H). This data demonstrates that deletion of *IFT80* in chondrocytes causes abnormal development in both growth plate and the articular cartilage.

### Loss of *IFT80* in chondrocytes disrupted cilia formation

**Fig 6:**
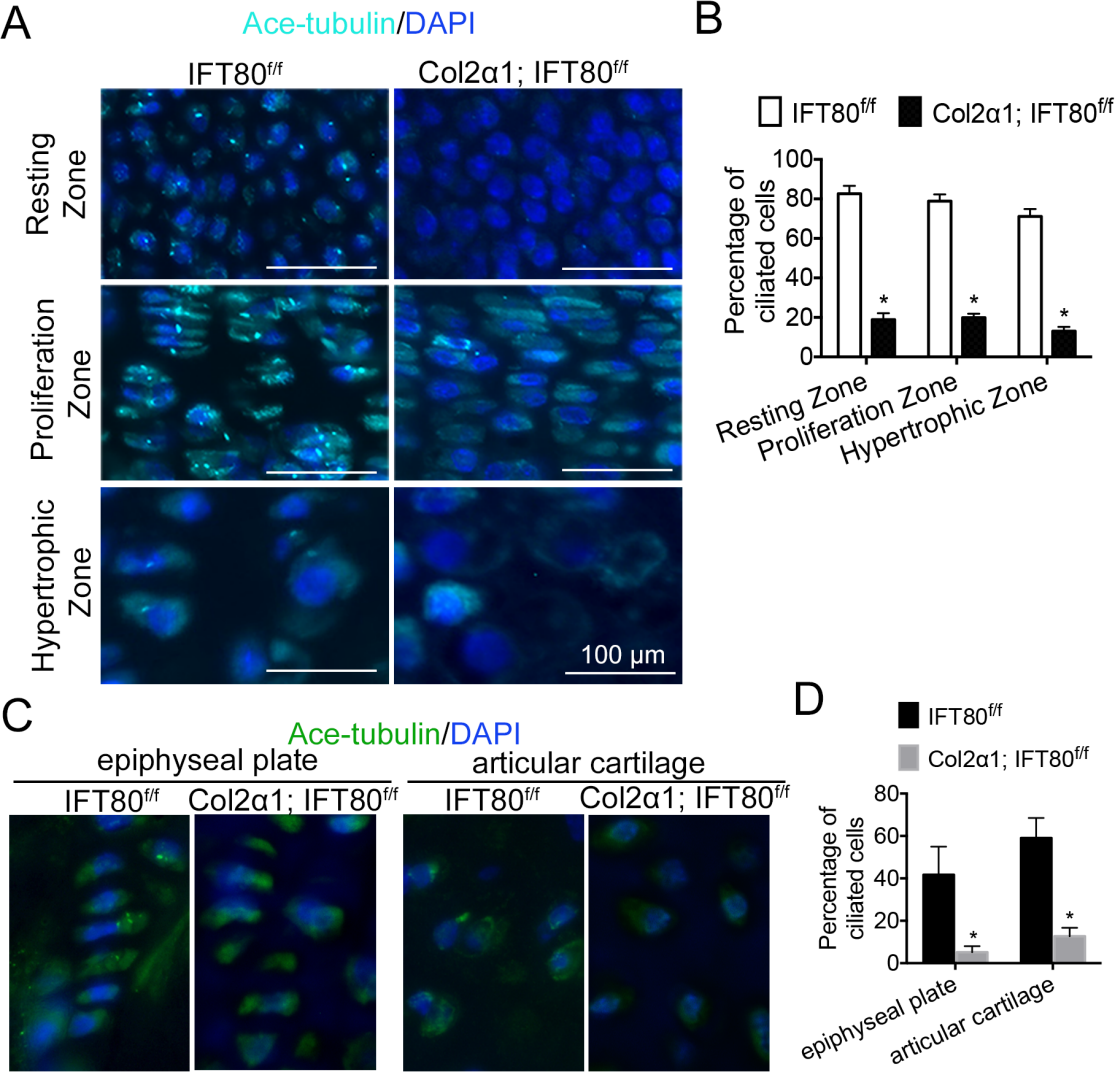
Significant cilia formation defects in the growth plate of *Col2α1; IFT80* ^*f/f*^ mice. (A) Immunofluorescence analysis of primary cilia in the tibial growth plate of newborn mice exposed to tamoxifen at E14.5, E16.5, and E18.5 (n = 3). Immunostaining of primary cilia was performed with acetylated α-tubulin (axoneme, cyan) antibody. DAPI (nuclear marker) staining was used as counterstain. Scale bars represent 100 μm. (B) Quantification of the ciliated cell population in resting zone, proliferation zone and hypertrophic zone (n = 3, *p<0.0001, significantly different from the *IFT80* ^*f/f*^ group). (C) Immunofluorescence analysis of primary cilia in the cartilage of P30 mice exposed to tamoxifen from P4-P7 and P14-P17 (n = 3). (D) Quantification of the ciliated cell population in epiphyseal plate and articular cartilage (n = 3, *p<0.0001, significantly different from the *IFT80* ^*f/f*^ group).

To further get insight into the mechanism of IFT80 in chondrocyte differentiation, we first performed cilia staining in the tibia section of the newborn mice, which were administered tamoxifen at 14.5, 16.5, and 18.5 days postcoitus. As shown in Fig 6A and 6B, about 80% chondrocytes in the resting zone and proliferation zone, and 70% in hypertrophic zone had normal cilia structure in *IFT80* ^*f/f*^ mice group. However, only about 20% chondrocytes in those zones showed cilia in *Col2α1; IFT80* ^*f/f*^ mice group (Fig 6A and 6B). Consistent to the results from the newborn mice, in *IFT80* ^*f/f*^ mice at postnatal day 30, 40% chondrocytes in epiphyseal plates and 60% in articular cartilage had cilia (Fig 6C and 6D). In contrast, cilia were absent in the growth plates and only present in 10% articular cartilage cells in *Col2α1; IFT80* ^*f/f*^ mice (Fig 6C and 6D).

### Loss of *IFT80* inhibited chondrogenic differentiation and chondrogenic marker gene expression

**Fig 7:**
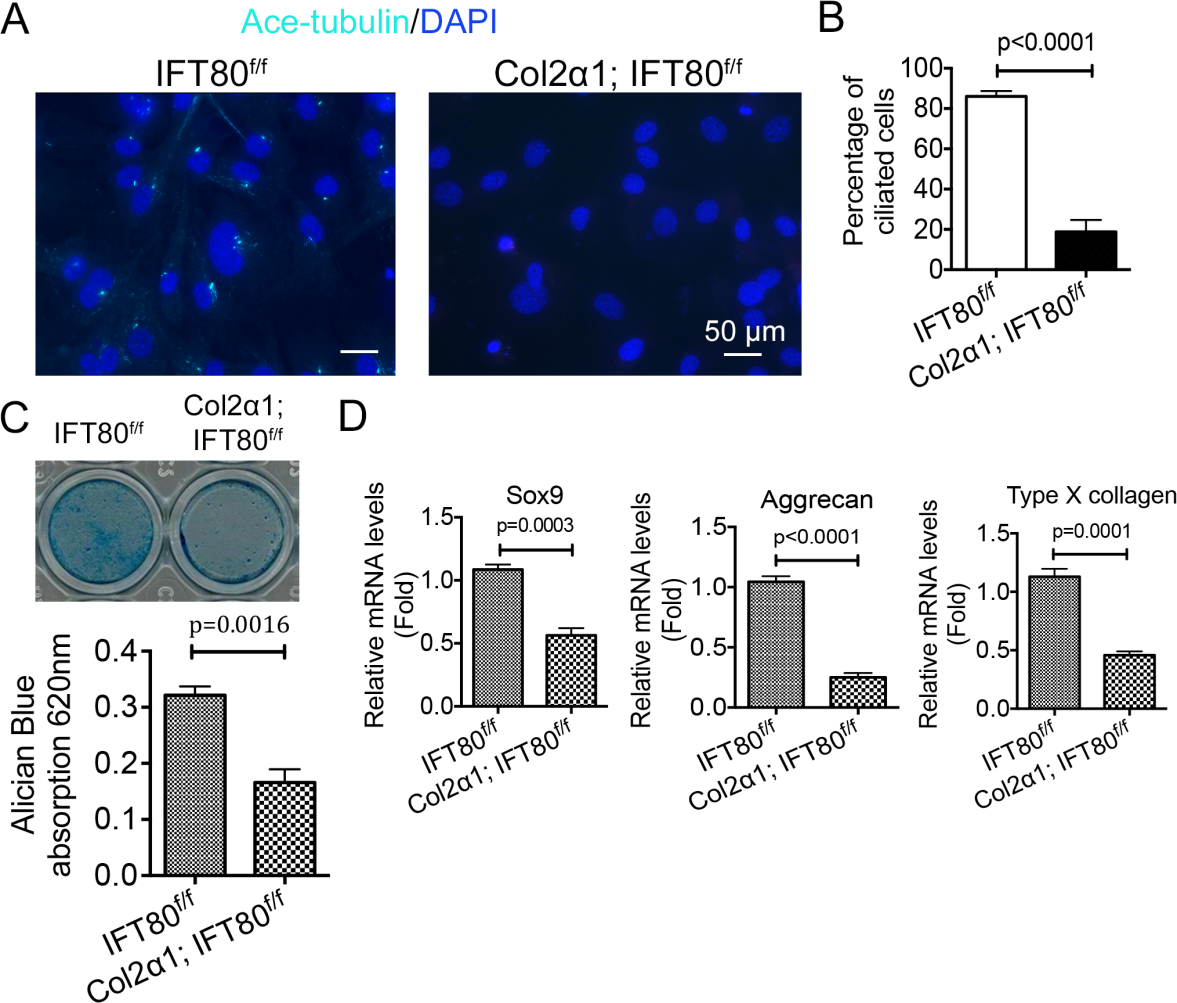
Deletion of *IFT80* in chondrocytes caused cilia loss and defects in chondrogenic differentiation. (A) Immunofluorescence analysis of primary cilia in chondrocytes derived from P10 *IFT80* ^*f/f*^ and *Col2α1; IFT80* ^*f/f*^ mice, which were administered tamoxifen at P4-7. (n = 4). Primary cilia were stained with acetylated α-tubulin (axoneme, cyan) antibody. DAPI (nuclear marker) staining was used as counterstain. Scale bars represent 50 μm. (B) Quantification of the ciliated cell population in chondrocytes derived from *IFT80* ^*f/f*^ and *Col2α1; IFT80* ^*f/f*^ mice (n = 4). (C) Proteoglycan production was assessed with Alcian blue staining at day 21 after chondrogenic induction. Quantitative assessment of Alcian blue staining was performed by measuring the optical density of the dyes extracted by 6 M guanidine-HCl (n = 3). (D) Chondrocyte marker genes expression profiles by qPCR. *Col2α1; IFT80* ^*f/f*^ chondrocytes in chondrogenic medium showed significantly lower expression of *Sox9*, *Aggrecan*, and *type X collagen* (n = 3).

To further investigate whether chondrodysplasia phenotype results from the effect of *IFT80* deletion on cilia formation and chondrogenic differentiation, chondrocytes were isolated from the cartilage of tamoxifen-injected *IFT80* ^*f/f*^ and *Col2α1; IFT80* ^*f/f*^ mice as described in Methods. We found that chondrocytes derived from *Col2α1; IFT80* ^*f/f*^ and cultured *in vitro* also showed reduced ciliated population compared to chondrocytes derived from *IFT80* ^*f/f*^ mice after 48 hours of serum starving, confirming the critical role of IFT80 in cilia formation (Fig 7A and 7B). To investigate the role of IFT80 in chondrogenic differentiation, chondrocytes derived from *IFT80* ^*f/f*^ and *Col2α1; IFT80* ^*f/f*^ were induced with chondrogenic medium for 21 days. Alcian blue staining was used to detect sulfated proteoglycan deposits, indicating functional chondrocytes. *Col2α1; IFT80* ^*f/f*^ chondrocytes displayed significantly less sulfated proteoglycan deposits compared to *IFT80* ^*f/f*^ chondrocytes (Fig 7C). Consistent with the defects in chondrogenic differentiation, *Col2α1; IFT80* ^*f/f*^ chondrocytes displayed reduced expression of the chondrocyte marker genes, including *Sox9*, *Aggrecan*, and *type X collagen* (Fig 7D).

### Loss of *IFT80* impaired Hh signaling activity but over-activated Wnt signaling activity

**Fig 8:**
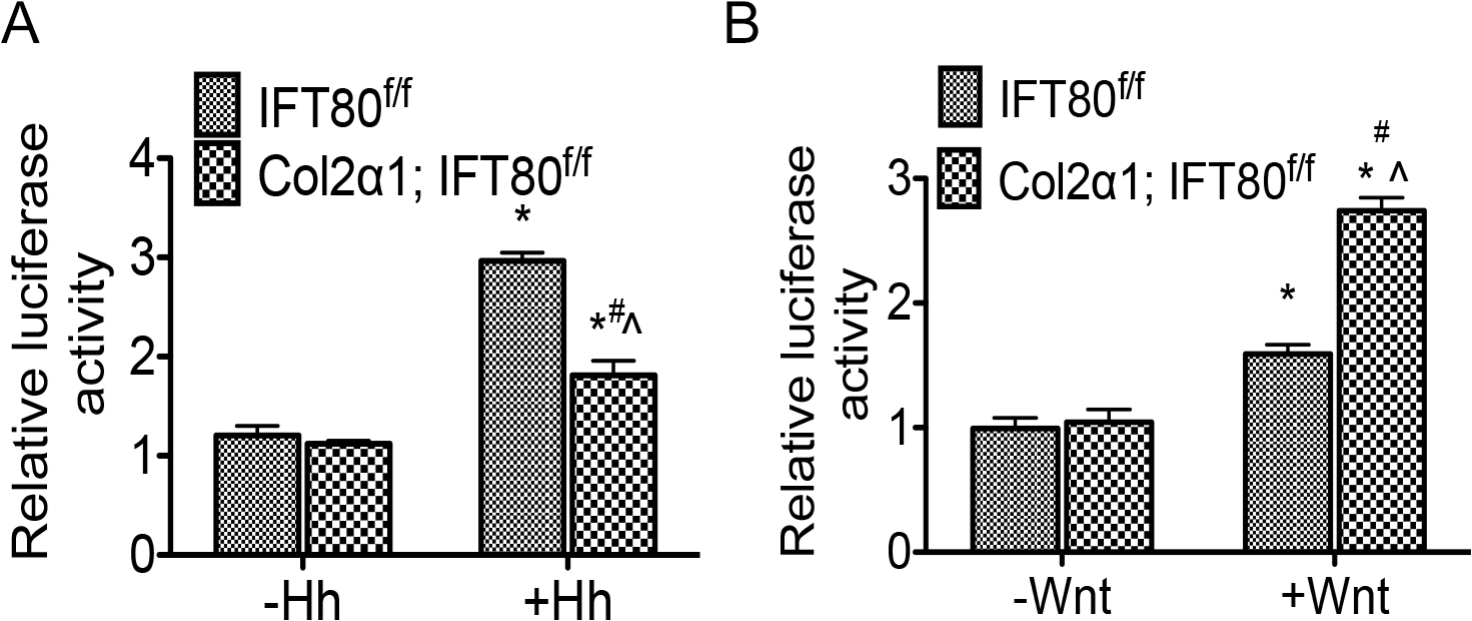
Deletion of *IFT80* in chondrocytes caused impaired Hh signaling, and enhanced Wnt signaling activity. Primary chondrocytes were derived from P10 *IFT80* ^*f/f*^ and *Col2α1; IFT80* ^*f/f*^ mice, which were administered tamoxifen at P4-7. (A) Reporter assay showing Gli responsive luciferase (8×Gli-Luc) activity. Shh (1 μg/mL) treatment resulted in significant increases in luciferase activity in *IFT80* ^*f/f*^ chondrocytes, whereas less stimulation with Shh was observed in the *Col2α1; IFT80* ^*f/f*^ chondrocytes (n = 3). (B) Wnt luciferase reporter assay with or without Wnt3a stimulation. 100 ng/mL Wnt3a significantly promoted higher luciferase activity in *Col2α1; IFT80* ^*f/f*^ chondrocytes compared to *IFT80* ^*f/f*^ chondrocytes (n = 3).

To gain further insight into the molecular mechanisms, we characterized the activation of Hh and Wnt signaling pathways, which are regulated by cilia or cilia related proteins [36, 37]. By performing a reporter assay using a p8×Gli-Luc construct [33], we found that stimulation with Shh resulted in a 3-fold increase in the Gli-responsive luciferase activity in *IFT80* ^*f/f*^ chondrocytes (Fig 8A). However, this activity significantly decreased in *Col2α1; IFT80* ^*f/f*^ chondrocytes (Fig 8A), indicating that Hh signaling activity is impaired. We also studied the role of IFT80 in Wnt signaling by performing a reporter assay with the M50 Super 8×TOPFlash construct. Wnt3a led to about 1.5-fold increase in luciferase activity in *IFT80* ^*f/f*^ chondrocytes, but almost 3-fold increase was found in *Col2α1; IFT80* ^*f/f*^ chondrocytes (Fig 8B), suggesting that loss of *IFT80* promotes Wnt signaling pathway transduction.

## Discussion

In the current study, we have demonstrated for the first time that IFT80 plays an essential role in embryonic epiphyseal and articular cartilage formation and maintenance of postnatal growth plate and bone formation. Previous studies have shown that mice with deletions in some other IFT genes such as IFT88 in chondrocytes at embryonic stage displayed the similar phenotype. Our present study emphasizes that the expression of IFT80 in chondrocytes in embryonic or postnatal stage is required for cilia formation, chondrocyte differentiation, and growth plate development and maintenance by regulating Hh and Wnt signaling transduction.

In this study, we used *Col2α1-CreER* line to study the function of IFT80 in cartilage development in both embryonic and postnatal stages. *Col2α1-CreER* is an inducible Cre line, in which Cre recombinase activity is controlled by the injection of tamoxifen in a time-specific manner [22]. Cre recombinase activity of *Col2α1-CreER* line could be first detected 8 hours post tamoxifen injection and extensive recombinase activity was found 24 hours post injection [22, 38]. Type II collagen has been reported expressed mainly in cartilage and have certain low level of expression in some other tissues including skin and kidney [39]. Cre expression driven by Col2α1 promoter is mainly found in perichondrium, which subsequently gives rise to bone [40, 41]. *Col2α1-CreER* line we used here has been proved to avoid Cre expression in osteogenic lineage by injection of tamoxifen after E13.5, and tamoxifen injection before E13.5 would target perichondrium [22]. Maeda et al. also studied the cartilage-specificity of this *Col2α1-CreER* line [42]. By crossing *Col2α1-CreER* line with *Rosa26; Cre* mice (expressing LacZ to indicate Cre activation) and injecting tamoxifen at postnatal day 0 or day 14, they found that LacZ expression is very specific in cartilaginous areas in different tissues, including ribs, paws, skull and sternum [42]. In our study, we began tamoxifen injection at E14.5 to avoid deleting *IFT80* in osteogenic lineage. Another notable fact of this *Col2α1-CreER* line is that Cre recombinase activity begins to decline after postnatal day 21, and is almost undetectable in 12-week-old mice [22, 38]. So in this study, we harvested our mutant mice within postnatal 30 days to avoid inefficient deletion of *IFT80*.

In the current study, tamoxifen-induced *IFT80* deletion in chondrocytes led to the dwarfism with reduced growth plate length (Figs 4 and 5). Loss of *IFT80* resulted in cilia loss and blocked chondrocyte differentiation (Fig 7), which may contribute to the reduction of the growth plate in *Col2α1; IFT80* ^*f/f*^ mice. Previous study showed that *Tg737* ^*orpk*^ (ORPK) mice, carrying a hypomorphic mutation in *IFT88*, also displayed smaller growth plates [43]. Song *et al*. found deletion of *IFT88* with *Col2α-Cre* caused post-natal dwarfism with premature loss of growth plates [44]. Both IFT88 and IFT80 are the core protein in the IFT complex B, and deletion *IFT88* or *IFT80* resulted in cilia loss, so we cannot rule out the possibility that the cartilage abnormality observed in this study is due to ciliary dysfunction. However, it is worth mentioning that the ciliopathies caused by different IFT protein mutations have different clinical abnormalities [5, 45], implying that not all IFT proteins only function through cilia formation. Most notably, in the *IFT80* gene-trap line generated by Rix et al, *IFT80* is expressed in a low level, which does not affect normal cilia formation [21]. But still, these mutation mice show disorganization of growth plate layers and shortened long bone. These findings indicate that, IFT80 most likely has its unique function(s) other than only builds up cilia. Loss of cilia, resulted from *IFT80* mutation, may be one of the causes for the phenotype. Interestingly, *IFT80* gene-trap line also showed constricted thoracic cages, which mimics JATD and SRP patients [21]. However, we did not observe significant thoracic constriction in the *Col2α1; IFT80* ^*f/f*^ mice. It is possible that IFT80 functions in both bone and cartilage to regulate rib development, whereas in this study, we avoided to delete *IFT80* in osteogenic lineage, and therefore rib development could only be slightly affected. Besides cartilage defect, we also found the trabecular bone loss in *Col2α1; IFT80* ^*f/f*^ mice. This may be caused by the failure in the generation of enough mature chondrocytes, and therefore insufficient production of extracellular matrix needed to support osteoblast differentiation and bone development. It is also possible that the lack of direct Hh signaling from chondrocytes to osteoblasts blocked bone formation.

Unlike epiphyseal cartilage, articular cartilage is a permanent cartilage and functions throughout postnatal life. Homeostasis of articular cartilage is important for cartilage integrity and joint function. The rate of cell differentiation and matrix turnover are extremely low in normal articular cartilage, but little is known about how these processes are regulated [46, 47]. In our study, we observed an increase of the thickness and cell density of the articular cartilage of *Col2α1; IFT80* ^*f/f*^ mice (Fig 5D and 5D’). This is in agreement with previous study that *Col2aCre;Ift88* ^*fl/fl*^ mice showed increased thickness and cell density within the articular cartilage layer due to reduced cell apoptosis [36]. These suggest that IFT proteins are also involved in the articular cartilage homeostasis. Moreover, *Col2aCre;Ift88* ^*fl/fl*^ mice showed alterations in the shape of the knee joint. However, the morphology of the knee joint was maintained in our *Col2α1; IFT80* ^*f/f*^ mice, and the altered articular cartilage phenotype was milder than that of *Col2aCre;Ift88* ^*fl/fl*^ mice. This is likely because *IFT80* was only deleted for a short period time, while *IFT88* was inactivated in knee joint for longer time since embryonic stage. It is also possible that IFT80 and IFT88 have distinct functions in the articular cartilage, which needs further studies.

Our results demonstrated that IFT80 plays a key role in Hh signaling transduction. *Col2α1; IFT80* ^*f/f*^ mice have similar phenotype to those of *Col2aCre; Ihh* ^*d*^ */Ihh* ^*d*^ and *Col2a-CreER; IHH* ^*d/d*^ mice [42, 48], showing stunted growth with shortened growth plate. Ihh directly regulates the differentiation of chondrocytes [49]. Therefore, these findings support our findings that deletion of IFT80 blocked Gli2 activity and Hh signaling in *Col2α1; IFT80* ^*f/f*^ mice. It can be reasonably explained that deletion of *IFT80* in chondrocytes causes cilia loss, which impairs Hh signaling transduction by inhibiting smoothen to translocate to cilia to activate Gli2 based on our data (Fig 8A) and other studies [50, 51], and this eventually caused the defects of chondrocyte differentiation. However, It is also possible that deletion of IFT80 not only affects Hh signaling pathway through cilia dependent pathway, but also through cilia independent direct functional pathway. Because the expression of lower level *IFT80* in IFT80 gene-trap line does not affect cilia formation, but disrupts Hh signaling, demonstrating IFT80 likely also regulates Hh signaling through cilia independent pathway [21].

Studies have suggested that Wnt/β-catenin signal is another signaling pathway highly involved in chondrocyte differentiation, growth plate assembly, and cartilage integrity [52–55]. Inhibition of β-catenin degradation blocked chondrogenesis [56] and expression of stabilized β-catenin in chondrocytes led to severe chondrodysplasia and dramatic inhibition of chondrocyte differentiation [57], suggesting that Wnt/β-catenin signaling is under strict regulation during chondrocyte differentiation and excessive signaling activity caused chondrogenesis defects. In good agreement of these studies, we found that Wnt reporter activity was significantly increased in *IFT80* deleted chondrocytes (Fig 8B), which may block the chondrocyte differentiation and maturation process. In addition, tamoxifen induced β-catenin activation in cartilage reduced the growth plate length (especially the hypertrophic zone) with loss of columnar alignment of chondrocytes two weeks after injection [58]. Consistent with this report, we found the short growth plate with unorganized chondrocytes (Figs 2 and 5) and reduced proteoglycans (Figs 2B’, 5D’, 5F’ and 7C) and aggrecan (Fig 7D) expression in *Col2α1;IFT80* ^*f/f*^ mice. In support of these findings, Chang et al found the aberrant Hh and Wnt signaling transduction in *Col2αCre;Ift88* ^*fl/fl*^ mice [59]. They found that defect in Hh signaling reduced the expression of Sfrp5, an extracellular antagonist of Wnt signaling pathway, and subsequently up-regulated Wnt signaling [59]. Taken these together, deletion of *IFT80* causes cilia loss and alters both Hh and Wnt signaling, leading to abnormal cartilage development in *Col2α1; IFT80* ^*f/f*^ mice.

In summary, our findings demonstrate that IFT80 plays an important role in the development and maintenance of both the epiphyseal and the articular cartilage, probably through controlling chondrocyte differentiation, Hh signaling and Wnt signaling pathways. Mutation of *IFT80* causes JATD and SRP with severe cartilage abnormalities. Our findings regarding the regulation of IFT80 in chondrocyte differentiation, cartilage formation, and Hh and Wnt signaling pathways, may open a new path to treat bone diseases including JATD, SRP, osteoarthritis by manipulating either IFT80 expression or related signaling pathways.

## Supporting Information

S1 Fig *Col2α1-Cre*-mediated conditional deletion of *IFT80* from the floxed *IFT80* allele (*IFT80* ^*flox*^). (A) Schematic illustration of the wild type allele, floxed *IFT80* allele (*IFT80* ^*flox*^) and *IFT80* mutant (*Col2α1; IFT80* ^*f/f*^). The targeting vector contains a 5.59 kb left arm homology, a Frt-flanked Neo reporter gene and LacZ gene, a loxp-flanked exon 6 of *IFT80*, and a 4 kb right arm homology. *IFT80* ^*LacZNeoFlox*^ mice were first mated with FLP transgenic mice to delete *neo* and *LacZ* to generate *IFT80* ^*flox*^ alleles. Deletion of the loxp cassette was achieved by *Col2α1* ***-*** *Cre*-mediated recombination.
(TIF)

S2 Fig Hematoxylin and eosin staining showing the trabecular bone of tibia from newborn *IFT80* ^*f/f*^ and *Col2*α*1; IFT80* ^*f/f*^ mice (received tamoxifen at E14.5, E16.5, and E18.5). SI_Caption>
(TIF)
