# Supplementary figures and images for: Deletion of IFT80 Impairs Epiphyseal and Articular Cartilage Formation Due to Disruption of Chondrocyte Differentiation
Source: PLoS One. 2015 Jun 22;10(6):e0130618. doi: 10.1371/journal.pone.0130618 (PMC4476593; doi:10.1371/journal.pone.0130618)

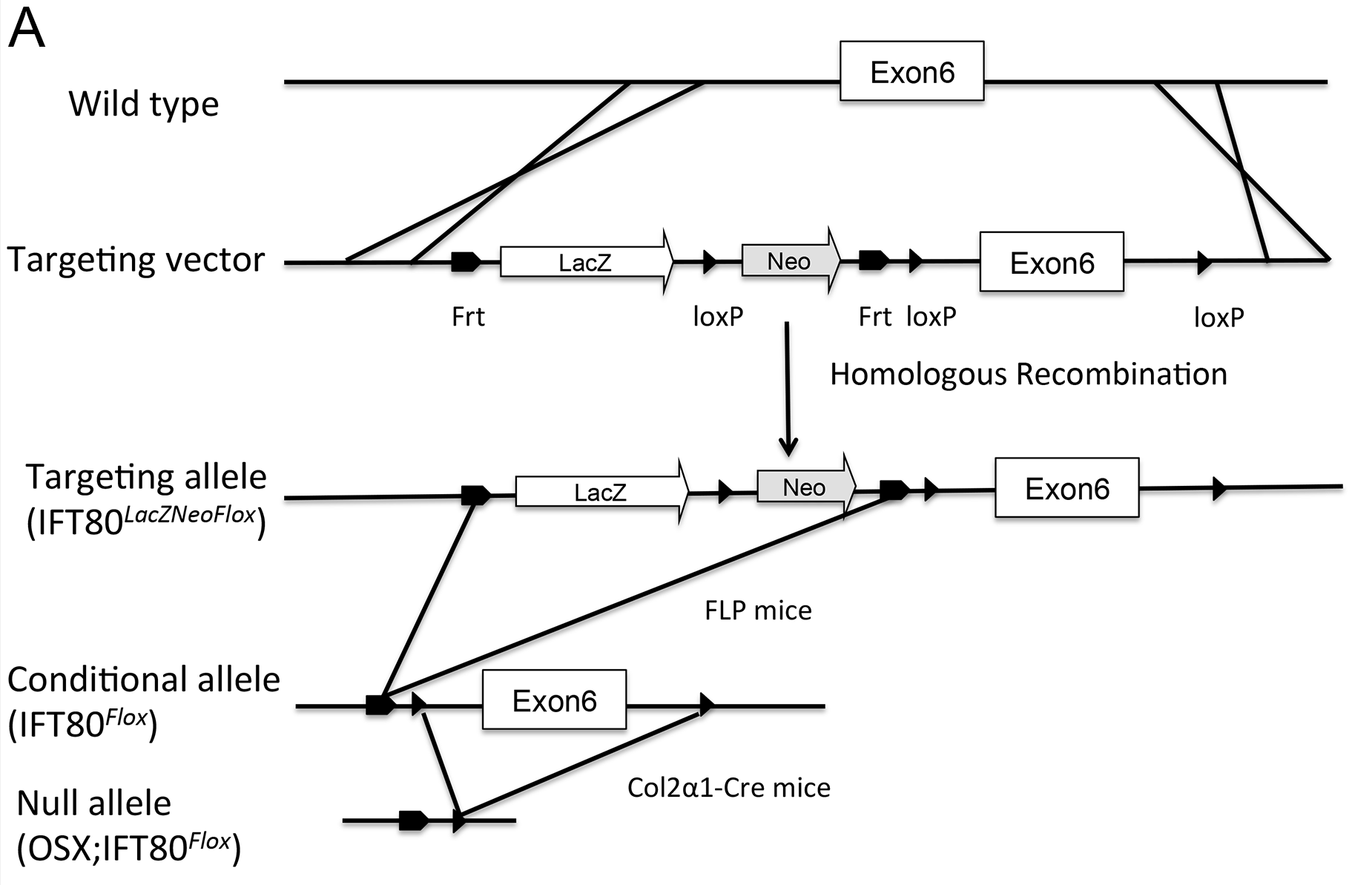

Supplement: S1 Fig — (A) Schematic illustration of the wild type allele, floxed IFT80 allele (IFT80 flox) and IFT80 mutant (Col2α1; IFT80 f/f). The targeting vector contains a 5.59 kb left arm homology, a Frt-flanked Neo reporter gene and LacZ gene, a loxp-flanked exon 6 of IFT80, and a 4 kb right arm homology. IFT80 LacZNeoFlox mice were first mated with FLP transgenic mice to delete neo and LacZ to generate IFT80 flox alleles. Deletion of the loxp cassette was achieved by Col2α1 - Cre-mediated recombination. (TIF) [file pone.0130618.s001.tif]

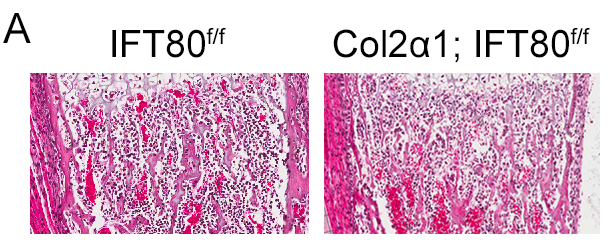

Supplement: S2 Fig — SI_Caption> (TIF) [file pone.0130618.s002.tif]
